# Ciprofloxacin Resistance and Gonorrhea Incidence Rates in 17 Cities, United States, 1991–2006

**DOI:** 10.3201/eid2004.131288

**Published:** 2014-04

**Authors:** Harrell W. Chesson, Robert D. Kirkcaldy, Thomas L. Gift, Kwame Owusu-Edusei, Hillard S. Weinstock

**Affiliations:** Centers for Disease Control and Prevention, Atlanta, Georgia, USA

**Keywords:** drug resistance, Neisseria gonorrhoeae, bacteria, gonorrhea incidence rates, drug therapy, epidemiology, ciprofloxacin resistance, cities, United States

## Abstract

Antimicrobial drug resistance can hinder gonorrhea prevention and control efforts. In this study, we analyzed historical ciprofloxacin resistance data and gonorrhea incidence data to examine the possible effect of antimicrobial drug resistance on gonorrhea incidence at the population level. We analyzed data from the Gonococcal Isolate Surveillance Project and city-level gonorrhea incidence rates from surveillance data for 17 cities during 1991–2006. We found a strong positive association between ciprofloxacin resistance and gonorrhea incidence rates at the city level during this period. Their association was consistent with predictions of mathematical models in which resistance to treatment can increase gonorrhea incidence rates through factors such as increased duration of infection. These findings highlight the possibility of future increases in gonorrhea incidence caused by emerging cephalosporin resistance.

Each year, the estimated 820,000 incident cases of gonorrhea in the United States result in lifetime direct medical costs of $162 million (*1*,*2*). Although substantial, the incidence of gonorrhea in the United States has decreased since the 1970s in part because of sexually transmitted disease (STD) prevention programs (*3*–*5*). However, treatment and control efforts can be hindered by antimicrobial drug resistance (*6*–*8*).

*Neisseria gonorrhoeae* has been remarkably adept at acquiring and maintaining resistance to antimicrobial drugs used for treatment, such as penicillin, tetracyclines, and fluoroquinolones (e.g., ciprofloxacin). After first spreading in Hawaii and California during the late 1990s and early 2000s, ciprofloxacin-resistant gonococcal strains became increasingly prevalent in the United States during the 2000s. By 2007, the Centers for Disease Control and Prevention (CDC) no longer recommended ciprofloxacin or other fluoroquinolones for treatment of gonorrhea, which make the cephalosporins cefixime or ceftriaxone the only remaining recommended treatment option (*9*).

During the past several years, gonococcal susceptibility to the cephalosporins has been decreasing (*6*–*8*). In response to increasing cefixime MICs in the United States, CDC recently updated its treatment recommendations for gonococcal infections (*10*). CDC now recommends dual therapy with ceftriaxone (an injectable cephalosporin) and a second antimicrobial drug as the only remaining recommended first-line treatment option for gonorrhea (*10*). However, the possible emergence and spread of cephalosporin resistance could eventually threaten the effectiveness of this regimen and pose a major public health challenge (*6*–*8*).

Although the course of emerging cephalosporin resistance and the possible effect on gonorrhea incidence are difficult to predict, it is possible to analyze historical trends in gonorrhea incidence during periods of increasing resistance to previously recommended antimicrobial drugs. In this study, we analyzed historical ciprofloxacin resistance data and gonorrhea incidence data to examine the possible effect of antimicrobial drug resistance on gonorrhea incidence at the population level. Assessing the historical population-level association between ciprofloxacin resistance and gonorrhea incidence can provide information about cephalosporin resistance in the future.

## Methods

### Overview

We first focused on simple comparisons of trends in gonorrhea incidence rates in 2 groups of cities in the United States: those with relatively high prevalence and those with relatively low prevalence of ciprofloxacin resistance. After performing these illustrative comparisons, we used regression analyses to examine the association between ciprofloxacin resistance and gonorrhea incidence in a more robust manner. For simplicity, we describe our study as a city-level analysis, although as described in more detail below, the data we analyzed comprised a mixture of sources at the city, county, and metropolitan statistical area levels.

### Gonococcal Isolate Surveillance Project

We used antimicrobial drug susceptibility data from the Gonococcal Isolate Surveillance Project (GISP) to analyze the association between ciprofloxacin resistance and gonorrhea incidence over time at the city level. GISP has been described in detail by Schwarcz et al. (*11*) and Kirkcaldy et al. (*12*). In brief, GISP is a sentinel surveillance system established in 1986 to monitor antimicrobial drug susceptibility among *N. gonorrhoeae* isolates. Each month, urethral gonococcal isolates and clinical data are systematically collected consecutively from up to the first 25 men at participating STD clinics in each city in whom urethral gonorrhea was diagnosed (*11*,*12*). Cities may have a single participating clinic or multiple clinics. The gonococcal isolates are tested for antimicrobial drug susceptibility by using agar dilution method. Ciprofloxacin susceptibility has been monitored in GISP since 1990 (*9*). The prevalence of ciprofloxacin resistance increased during the late 1990s and 2000s (*9*). By 2007, ciprofloxacin resistance was prevalent in all regions of the United States, prompting CDC to no longer recommend fluoroquinolones for treatment of gonorrhea (*9*). Given this time line and availability of data for additional variables described later, we included the years 1991–2006 in our analysis.

Cities with ≥1 STD clinic participating in GISP were included in the study if annual ciprofloxacin resistance prevalence data were available from that city for ≥13 years during 1991–2006 and if city-level gonorrhea incidence rates during the same 16-year period were available from gonorrhea case report surveillance data maintained by the Division of STD Prevention at CDC. Seventeen cities met the inclusion criteria: Albuquerque (New Mexico), Atlanta (Georgia), Baltimore (Maryland), Birmingham (Alabama), Cincinnati (Ohio), Cleveland (Ohio), Denver (Colorado), Honolulu (Hawaii), Minneapolis (Minnesota), New Orleans (Louisiana), Philadelphia (Pennsylvania), Phoenix (Arizona), Portland (Oregon), San Diego (California), San Francisco (California), Seattle (Washington), and St. Louis (Missouri). For most cities in our analysis, the city-specific STD rates we obtained were derived from county data and may only approximate city jurisdictions. Our dataset consisted of 272 observations, and each observation included the annual prevalence of city-level gonococcal ciprofloxacin resistance (prevalence in 17 cities each year over a 16-year period).

### Gonorrhea Incidence Rates in Cities Grouped by Ciprofloxacin Resistance

We calculated the median percentage of isolates resistant to ciprofloxacin in 2004 and labeled the 8 cities above the median as higher resistance cities and the 9 cities at or below the median as lower resistance cities. For each group, we calculated gonorrhea incidence rates during 1991–2006. The rate for each group of cities was calculated as the sum of reported gonorrhea cases in the cities divided by the sum of the populations of the cities and multiplied by 100,000. The percentage of isolates resistant to ciprofloxacin for each group of cities was calculated as the average across all cities in the group.

### Regression Analyses—Description of Data and Model Overview

We performed regression analyses in which the dependent variable was the city gonorrhea incidence rate (log) and the independent variable of interest was the percentage of GISP isolates resistant to ciprofloxacin in GISP clinic(s) located in the given city. The regression also included sociodemographic variables (percentage of persons who were black, percentage of persons 15–29 years of age, unemployment rate, per capita income, robbery rate) and binary (dummy) variables for each city and year to control for city-specific factors and national trends in factors that influence city-level gonorrhea incidence rates (Table 1).

**Table 1:** Variables used in regression analyses of ciprofloxacin resistance and gonorrhea incidence rates in 17 cities, United States, 1991–2006*

Variable	Mean (SD)	Description	Source
Ciprofloxacin resistance	0.028 (0.070)	Fraction of GISP isolates resistant to ciprofloxacin (MIC ≥1 μg/mL) in GISP clinic(s) in given city	GISP
Gonorrhea incidence rate (log)	5.60 (0.911)	Log of city’s reported gonorrhea incidence rate (cases/100,000 persons)	CDC
Syphilis rate (log)	2.07 (1.22)	Log of city’s reported primary and secondary syphilis rate (cases/100,000 persons)	CDC
% Black	24.3 (21.6)	% of city population that is black	Census
% 15–29 y of age	21.7 (1.6)	% of city population that is 15–29 y of age	Census
Robbery rate	589 (356)	No. reported offenses/100,000 persons	FBI
Unemployment rate	6.07 (1.99)	% of city’s labor force not employed	BLS
Per capita income	$36,483 ($5,788)	Per capita personal income in the city’s respective metropolitan statistical area (2006 dollars)	BEA
City variables	NA	Binary (dummy) variables for each city	Created
Year variables	NA	Binary (dummy) variables for each year	Created

*For simplicity, we describe our study as a city-level analysis, although the data we analyzed were comprised of a mixture of sources at the city level, county level, and metropolitan statistical area. The dataset consisted of 1 observation/city/year during 1991–2006. Gonorrhea and syphilis incidence rates, % Black, and % 15–29 y of age were obtained from surveillance records and US Census Bureau data maintained by CDC (Atlanta, GA, USA) (*13*). We added 1 to the syphilis rate before taking the log. The city-specific data obtained from CDC were derived from county data and may only approximate city jurisdictions. City-specific resistance was based on resistance reported in GISP. Robbery rates and unemployment rates were based on city-level data, and per capita income was based on metropolitan statistical area data (www.ucrdatatool.gov, http://www.bls.gov/data and http://bea.gov/, respectively). Per capita income was updated to 2006 US dollars by using the all items component of the consumer price index (www.bls.gov/cpi/data.htm). For ease of display of regression coefficients, the unemployment rate was entered into the regression analyses as the no. persons unemployed/100 in the labor force, robbery rates were entered as the no. offenses/100 population, and income was entered in $100,000s (e.g., $36,483 was entered as 36.48). GISP, Gonococcal Isolate Surveillance Project; CDC, Centers for Disease Control and Prevention; FBI, Federal Bureau of Investigation; BLS, Bureau of Labor Statistics; BEA, Bureau of Economic Analysis.

We included percentage of persons who were black and percentage of persons 15–29 years of age as explanatory variables because reported STD rates are often disproportionately high among black persons and youth (*13*). We included unemployment, income, and robbery rates as explanatory variables because STD rates also have been linked to social determinants of health (*14*,*15*). Sociodemographic variables, such as these, have been shown to correlate with STD rates at the population level over time (*16*,*17*).

Gonorrhea and syphilis incidence rates, percentage of persons who were black, and percentage of persons 15–29 years of age were obtained from surveillance records and US Census Bureau data maintained by CDC (*13*). Robbery rates, unemployment rates, and per capita income data were obtained online from various federal agencies (Table 1). Of our 272 observations, 11 had missing values for ≥1 variable. Missing values for the variables were replaced with estimated values, and we assumed a linear trend from one year to the next. For example, if the unemployment rate for a given city in 2004 was missing, the average of the unemployment rate for the given city in 2003 and 2005 was assigned for 2004.

### Regression Model Details

A common problem with regression analysis of data consisting of multiple observations over time is serial correlation, in which the error term in a given year correlates with the error term in the previous year. We used 2 approaches to address the issue of serial correlation. First, we calculated SEs that are robust to the serial correlation. Second, we corrected for the autocorrelated error terms when computing the regression (*18*). Specifically, we used ordinary least squares (OLS), included the lagged dependent variable as an exploratory variable, and used the Newey-West procedure to calculate heteroskedasticity- and autocorrelation-consistent SEs for the regression coefficients. We also estimated a linear regression with correction for first-order autocorrelated errors (AR1) by using the AR1 procedure.

The specific equation we estimated with OLS was G_i,t_ = α + β_1_G_i,t-1_ + β_2_R_i,t_ + γX_i,t_ + C_i_ + Y_t_ + ε_i,t_, in which G_i,t_ is the log of the gonorrhea incidence rate in city *i* in year *t*, α is a constant, R_i,t_ is the percentage of isolates resistant to ciprofloxacin in GISP clinic(s) of city *i* in year *t*, X_i,t_ is a vector of sociodemographic variables listed earlier, C denotes city dummy variables, Y denotes year dummy variables, and ε is the error term. The equation we estimated with AR1 was the same as the previous equation except that the lagged value of the dependent variable (G_i,t – 1_) was not included in the model. Thus, the differences between the 2 approaches we used to address serial correlation can be summarized as follows. The OLS regression includes the lagged value of gonorrhea incidence rates as an independent variable and calculates SEs that are robust to autocorrelation in the error terms. The AR1 regression is corrected for first-order correlation in the error terms and does not include the lagged value of the gonorrhea incidence rate. Analyses were conducted by using WinRATS version 8.01 (Estima, Evanston, IL, USA).

### Additional Regression Analyses

We performed additional analyses to examine the robustness of our results. First, we repeated our regression analysis by substituting the log of the syphilis rate for the log of the gonorrhea incidence rate as the dependent variable, thereby testing to determine whether our model would suggest an implausible link between gonococcal ciprofloxacin resistance and changes in the incidence of syphilis. In performing this procedure, we added 1 to the syphilis rate before taking the log so as not to exclude observations in which the syphilis rate was 0. Second, we examined temporal aspects of the association between ciprofloxacin resistance and gonorrhea incidence rates to determine whether gonorrhea incidence rates could be better predicted on the basis of past values of gonorrhea incidence rates and ciprofloxacin resistance rather than past values of gonorrhea incidence rates alone (as in Granger causality tests) (*18*,*19*). To do so, we modified our model so that 3 lagged values of the resistance variable (R_i,t – 1_, R_i,t –2_, and R_i,t – 3_) were included as explanatory variables rather than the current year value of the resistance variable (R_i,t_). We also included 3 lagged values of gonorrhea incidence (specifically, the log of the gonorrhea incidence rate in years t – 3, t –2, and t –1) as explanatory variables rather than 1 lag. We examined the joint significance of the 3 lagged resistance variables (R_i,t – 1_, R_i,t – 2_, and R_i,t – 3_) by using an F test to compare this model with a restricted model in which the coefficients of these 3 variables were set to 0. The joint significance of the 3 lagged values of gonorrhea incidence was calculated in an analogous manner. We then reversed the model such that ciprofloxacin resistance was the dependent variable. Third, we tested the sensitivity of our results to functional form by using the gonorrhea incidence rate rather than the log of the gonorrhea incidence rate as the dependent variable. Fourth, we tested for the effect of influential observations by using 2 approaches: deleting observations with a residual >2 SEs and repeating the main analysis 17 times, each time omitting 1 of the 17 cities from the analysis.

## Results

The average fraction of GISP isolates resistant to ciprofloxacin across the 17 cities during the 16 years examined was 0.028 (Table 1) (range 0–0.445). The average logged value of the gonorrhea incidence rate was 5.6 cases/100,000 persons (Table 1), which corresponds to a rate of 270 cases/100,000 persons (range 49–2,265 cases/100,000 persons). The average city population was 24.3% black and 21.7% were 15–29 years of age (Table 1).

### Gonorrhea Incidence Rates in Cities with Ciprofloxacin Resistance

In 2004, a median percentage of 3.3% of isolates were resistant to ciprofloxacin in the 17 cities in our analysis. We classified the 8 cities above the median in 2004 as higher resistance cities and the 9 cities at or below the median in 2004 as lower resistance cities. Cities with higher resistance were Denver, Honolulu, Minneapolis, Phoenix, Portland, San Diego, San Francisco, and Seattle. Cities with lower resistance were Albuquerque, Atlanta, Baltimore, Birmingham, Cincinnati, Cleveland, New Orleans, Philadelphia, and St. Louis. In our simple comparison of higher resistance and lower resistance cities, we found divergent trends in gonorrhea incidence rates in the 2000s (Figure). Although gonorrhea incidence rates were much lower overall in the higher resistance cities, gonorrhea incidence rates generally increased in the higher resistance cities and decreased in the lower resistance cities during 2000–2006 (Figure, panel A). The timing of the divergent trends in gonorrhea incidence rates coincided with the divergent trends in ciprofloxacin resistance (Figure, panel B).

**Figure:**
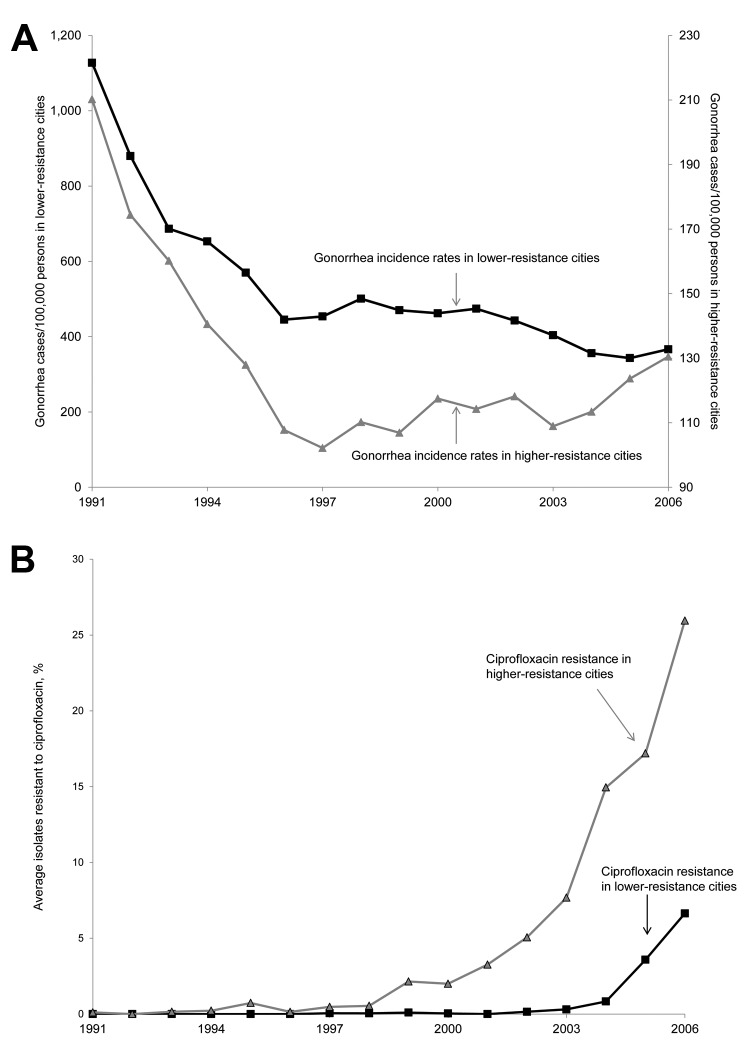
Ciprofloxacin resistance and gonorrhea incidence rates in 17 cities, United States, 1991–2006. A) Gonorrhea incidence rates and B) average percentage of isolates resistant to ciprofloxacin for 2 groups of cities with higher (above the median) and lower (at or below the median) percentages of isolates resistant to ciprofloxacin as of 2004. Cities with higher resistance were Denver (Colorado), Honolulu (Hawaii), Minneapolis (Minnesota), Phoenix (Arizona), Portland (Oregon), San Diego (California), San Francisco (California), and Seattle (Washington). Cities with lower resistance were Albuquerque (New Mexico), Atlanta (Georgia), Baltimore (Maryland), Birmingham (Alabama), Cincinnati (Ohio), Cleveland (Ohio), New Orleans (Louisiana), Philadelphia (Pennsylvania), and St. Louis (Missouri).

### Regression Analyses

The coefficient of the ciprofloxacin resistance variable was positive and significant across all 4 models we estimated (p<0.01) (Table 2). Ciprofloxacin resistance in a given city in a given year was associated with higher gonorrhea incidence rates in that city in the given year. This finding was consistent regardless of estimation procedure (OLS in models 1 and 2 and AR1 in models 3 and 4) and regardless of the exclusion (models 1 and 3) or inclusion (models 2 and 4) of the additional sociodemographic variables.

**Table 2:** Results of regression analysis of gonorrhea incidence rates in 17 cities, United States, 1991–2006*

Independent variable	Model 1	Model 2	Model 3	Model 4
Ciprofloxacin resistance	0.739 (0.172)†	0.710 (0.201)†	0.892 (0.322)†	0.926 (0.322)†
Lagged dependent variable	0.597 (0.052)†	0.553 (0.053)†	–	–
% Black	–	−0.143 (0.962)	–	0.991 (1.67)
% 15–29 y of age	–	−0.381 (1.20)	–	−1.60 (2.49)
Robbery rate	–	0.247 (0.058)†	–	0.336 (0.125)†
Unemployment rate	–	−0.660 (1.20)	–	−0.724 (1.83)
Per capita income	–	0.449 (0.656)	–	0.324 (1.19)
Adjusted R^2^	0.969	0.970	0.967	0.967

*Values are coefficients (SEs) unless otherwise indicated. All of the above regressions also included a constant term and binary (dummy) variables for city and year (not reported in table). Models 1 and 2 included the lagged value of the dependent variable and were estimated by using ordinary least squares. Models 3 and 4 were estimated by using linear regression corrected for first-order autocorrelated errors. –, variables were not included in the regression. †p<0.01.

### Additional Regression Analyses

We found no association between ciprofloxacin resistance and syphilis incidence. When we examined the temporal association between ciprofloxacin resistance and gonorrhea incidence, the coefficients of the lagged individual ciprofloxacin resistance variables were not all significant individually when the dependent variable was the log of the gonorrhea incidence rate (Table 3). However, the sum of the coefficients of the lagged ciprofloxacin resistance variables was positive and these coefficients were jointly significant (p<0.01). When we reversed the model such that ciprofloxacin resistance was the dependent variable, lagged values of the gonorrhea incidence coefficients (specifically the coefficients of the logs of the gonorrhea incidence rate in years t – 3, t – 2, and t – 1) were not jointly significant (Table 3). Although past levels of ciprofloxacin resistance helped to predict current gonorrhea incidence rates, past gonorrhea incidence rates did not help to predict current ciprofloxacin resistance levels. Our results were generally consistent across the range of additional analyses we conducted, including applying gonorrhea incidence rates in non-log form, omitting outliers, and omitting any given city from the analysis.

**Table 3:** Selected results of regression analyses of the temporal association of ciprofloxacin resistance and gonorrhea incidence rates in 17 cities, United States, 1991–2006*

Independent variable	Gonorrhea incidence rate (log), year t	Ciprofloxacin resistance rate, year t
Gonorrhea incidence rate (log), year t – 1	0.571 (0.057)†	0.015 (0.012)
Gonorrhea incidence rate (log), year t – 2	0.043 (0.080)	0.000 (0.012)
Gonorrhea incidence rate (log), year t – 3	−0.057 (0.077)	0.009 (0.010)
Ciprofloxacin resistance, year t – 1	−0.096 (0.488)	0.854 (0.154)†
Ciprofloxacin resistance, year t – 2	1.41 (0.538)†	0.395 (0.192)‡
Ciprofloxacin resistance, year t – 3	0.793 (0.492)	−0.127 (0.177)
Sum of gonorrhea incidence rate (log) coefficients	0.557 (0.070)	0.024 (0.014)
Joint significance of gonorrhea incidence rate (log) coefficients: F test	*F* = 46.6†	*F* = 1.09
Sum of ciprofloxacin resistance coefficients	2.11 (0.506)	1.12 (0.146)
Joint significance of ciprofloxacin resistance coefficients: F test	*F* = 8.88†	*F* = 66.7†
Adjusted R^2^	0.971	0.859

*Values are coefficients (SEs) unless otherwise indicated. Both of the above regressions also included a constant term and binary (dummy) variables for city and year (not reported in table) and were estimated by using ordinary least squares. These findings, that past levels of ciprofloxacin resistance helped to predict current gonorrhea incidence rates but that past gonorrhea incidence rates did not help to predict current ciprofloxacin resistance levels, were generally consistent when linear regression corrected for first-order autocorrelated errors was used rather than ordinary least squares and/or when including additional covariates (% Black, % 15–29 y of age, robbery rate, unemployment rate, and per capita income). †p<0.01. ‡p<0.05.

## Discussion

We found a strong positive association between ciprofloxacin resistance and gonorrhea incidence rates at the city level during 1991–2006. However, ecologic studies, such as ours, of the population-level association between ciprofloxacin resistance and gonorrhea incidence cannot establish that this association is causal. Nonetheless, our study offers evidence consistent with that of a causal association between drug resistance and increased incidence. In focusing on the temporal order of the association between ciprofloxacin resistance and gonorrhea incidence rates, we found a strong association between ciprofloxacin resistance and subsequent gonorrhea incidence rates. In contrast, we did not find a robust association between gonorrhea incidence rates and subsequent ciprofloxacin resistance. Nor did we did find an association between ciprofloxacin resistance and syphilis incidence. If the association we observed between ciprofloxacin resistance and gonorrhea incidence rates were spurious, we might also expect to find an association between ciprofloxacin resistance and syphilis incidence rates, given a strong association between syphilis rates and gonorrhea incidence rates among the cities in our analysis for most years during 1991–2006.

Although we found that ciprofloxacin resistance may have contributed to increases in gonorrhea incidence, reported gonorrhea incidence rates were generally lower in cities that had higher levels of ciprofloxacin resistance than in cities that had lower levels of ciprofloxacin resistance. Thus, any effect that increased ciprofloxacin resistance might have had on gonorrhea incidence rates during the late 1990s and early 2000s would likely be relatively minor compared with all other factors that influence gonorrhea incidence at the population level.

Our results can help to quantify the possible effect of antimicrobial drug resistance on the incidence of gonorrhea at the population level. In model 2, the resistance coefficient was 0.710, which suggested that a change of 0.1 in the resistance variable would be associated with an increase in gonorrhea of ≈7%. Thus, our findings suggest that gonorrhea incidence rates in a scenario in which 10% of isolates were resistant to treatment would be ≈7% higher than in a scenario of no drug resistance, although the cumulative effect of resistance over time could be more substantial.

At least 2 possible explanations exist for the observed association. First, treatment failures or delays in clearance of infections caused by ciprofloxacin resistance might have increased the duration of infectivity and facilitated transmission to partners. Second, mutational changes in the organism that conferred resistance or co-occurred with resistance determinants might have supported gonococcal transmission. This possibility is suggested in the study reported by Kunz et al. that mutant gyrase (gyrA)_91_*_/95_* alleles in *N. gonorrhoeae* appeared to provide fitness benefit (*20*).

Our assessment of the association between ciprofloxacin resistance and gonorrhea incidence offers evidence that emerging cephalosporin resistance could lead to higher gonorrhea incidence rates at the population level than would have been observed in the absence of cephalosporin resistance. However, because *N. gonorrhoeae* ciprofloxacin resistance might differ in several ways from cephalosporin resistance, the possible effect of cephalosporin resistance on gonorrhea incidence rates might differ substantially from that of ciprofloxacin resistance. Whereas ciprofloxacin resistance is conferred by amino acid substitutions in the A subunits of DNA gyrase and parC, the A subunit of DNA topoisomerase IV (*21*), cephalosporin resistance, particularly ceftriaxone resistance, may require acquisition of an unusual *penA* mosaic allele and mutations in *mtrR*, *penB*, and *ponA1* (*22*,*23*). The ease with which *N. gonorrhoeae* can acquire these resistance determinants is unclear, and the biologic fitness of ceftriaxone-resistant strains is unknown. During the emergence of ciprofloxacin resistance, non-fluoroquinolone treatment options were readily available. However, few, if any, alternative options are available to treat ceftriaxone-resistant infections. In this scenario of limited treatment options, the population-level effect of ceftriaxone resistance could be more substantial.

STD surveillance data are subject to limitations, such as incomplete reporting of cases and differences across jurisdictions in how data are collected (*13*). Furthermore, for most cities in our analysis, the city-specific STD rates we obtained were derived from county data and might only approximate data for city jurisdictions. However, our use of binary (dummy) variables for each city helps to guard against possible biases that arise because of constant differences across cities in STD reporting practices and the use of county-level data to approximate data for city jurisdictions. The sociodemographic variables we included might likewise only approximate those for city jurisdictions because some of these variables were based on county data and some were based on metropolitan statistical area data. However, biases in the sociodemographic variables are unlikely to have influenced our findings substantially because the association we observed between ciprofloxacin resistance and gonorrhea incidence was consistent regardless of whether the sociodemographic variables were included in the model.

We assumed that the ciprofloxacin resistance in isolates collected from STD clinic(s) in a given city in a given year reasonably represent resistance for the entire city in the given year. Although overall prevalence of gonorrhea in STD clinics is not representative of the overall population because STD clinic attendees are generally at higher risk, those infected with gonococcal infections with lower (or greater) antimicrobial drug susceptibility are unlikely to preferentially attend these clinics. Because our analysis was limited to cities for which GISP susceptibility data and city-level gonorrhea incidence were available, the cities in our study might not be representative of other US cities.

Although we controlled for sociodemographic factors, city effects, and year effects, we were unable to control for all city-specific factors that might influence gonorrhea incidence rates. For example, we were unable to control for city-specific changes in gonorrhea treatment regimens over time because of lack of data.

This study helps to quantify the association between ciprofloxacin resistance and gonorrhea incidence and can inform assessments of the possible effect of emerging resistance to current gonorrhea treatment. The association we observed is consistent with predictions of mathematical models in which resistance to treatment can increase gonorrhea incidence rates through factors such as increased duration of infection (*24*,*25*).

Ciprofloxacin resistance was associated with increases in gonorrhea incidence rates during the late 1990s and 2000s despite availability of other well-studied recommended treatment options. Correspondingly, emerging cephalosporin resistance could have even more substantial health and economic consequences, particularly as the number of available treatment options decreases. Efforts to control the spread of drug-resistant strains may mitigate this possible effect.
